# Social distancing: how religion, culture and burial ceremony undermine the effort to curb COVID-19 in South Africa

**DOI:** 10.1080/22221751.2020.1769501

**Published:** 2020-05-27

**Authors:** Ishmael Festus Jaja, Madubuike Umunna Anyanwu, Chinwe-Juliana Iwu Jaja

**Affiliations:** a Department of Livestock and Pasture Science, University of Fort Hare, Alice, South Africa; b Department of Veterinary Pathology and Microbiology, University of Nigeria, Nsukka, Nigeria; c Faculty of Medicine and Health Sciences, Department of Nursing and Midwifery, Stellenbosch University, Cape Town, South Africa

**Keywords:** Africa, tradition, burial ceremony, South Africa, SARS-CoV-2, COVID-19, religion

The 2019 novel coronavirus (2019-nCoV) has altered the way we live, interact and socialized. The viral infection first detected in Wuhan China has rapidly spread globally and subsequently declared a pandemic by the World Health Organization (WHO) [1,2]. As of the 7th of May 2020, the infection figures show 3,759,967 confirmed cases, 259,474 confirmed deaths in 215 countries, areas or territories [3]. In Africa, 53,609 cases have been reported, and 2061 deaths have been recorded [4]. South Africa has conducted 292,153 test, 8232 Positive Cases were Identified, 3153 cases have recovered, and 161 Deaths has so far been reported [5].

The novel coronavirus also called COVID-19, mainly affects the respiratory systems with catastrophic consequences in various body organs. The virulence and pathogenicity of COVID-19 are severe in the elderly and people with co-morbidity [6,7]. Many aspects of the new virus remain unclear; however, the clinical signs and pathogenesis of the disease have been described in many studies [1,8,9]. For instance, scientists are yet to confirm if humans previously exposed to the virus possess active immunity to prevent re-infection. Vaccines critical to preventing infections in humans are not yet available. For how long will the world remain under lockdown and when do we expect a return to times when things were normal?

Nineteen of the world's 20 youngest countries are in Africa; this youthful population offers a glimmer of hope as the impact of the virus may be limited [10]. However, the health care system and disease preparedness in the majority of the 54 African nations are virtually absent. Hence a large scale outbreak will be devastating not only to the older population but also to young people. This is precisely why almost all countries in Africa are under lockdown to stem the spread of COVID-19. Guidelines for social or physical distancing have been issued for essentials workers and other categories of people, who may wish to shop, use the taxi, visit the hospital, and attend burials.

**Figure 1.**
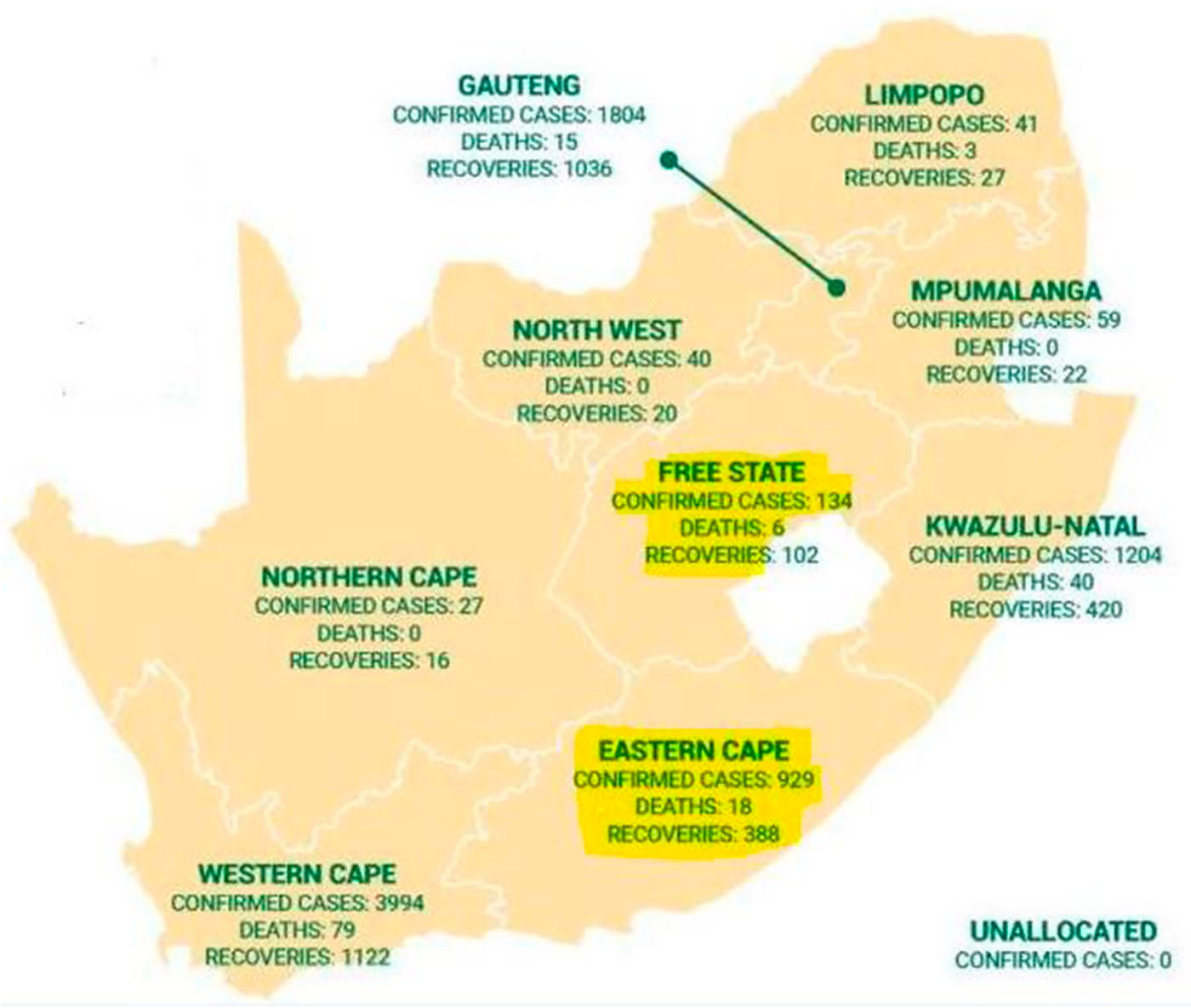
South Africa COVID-19 outbreak map, 7 May 2020. Church service and burial events in the two provinces highlighted in yellow lead to 80% of the COVID-19 cases. Source: SACOVID-19 portal [4] (https://sacoronavirus.co.za)

In a pandemic, social distancing measures have proven to be effective in reducing disease transmission [11]. However, Social distancing guidelines are not observed in many parts of South Africa; hence a spike in the infection rates in some provinces. For instance, in the Eastern Cape Province, 80% of all infections in the province resulted from burial ceremonies in Port St Johns, Port Elizabeth and Mthatha (Figure 1). The provincial Health Department issued a statement that in Port Elizabeth, 160 cases stemmed from two funerals. In Majola, Port St Johns, 40 new cases stemmed from one funeral, and in Mthatha, one case has also been linked to a funeral [12]. The COVID-19 infection incidence in the Eastern Cape Province as the 7th of May 2020 (Figure 1) has risen to over 900. The majority of the cases are linked to the three burial event.

Although the lockdown regulations allow a maximum of 50 attendees at a funeral, however, some families disobeyed this regulation, leading to a wave of new cases of COVID-19 infection in the province. During funerals, social distancing is not necessarily followed. For instance, food and water are shared, and people sit close to each other exchanging pleasantries. Also, cultural practice such as the washing of hands in one basin after the funeral presents an opportunity for guest to contract the virus. Furthermore, covering of the mouth using mask has not been strictly applied, hence singing is at funerals is another opportunity for the spread of droplets containing the virus.

The COVID-19 outbreak in the Free State follows a similar pattern after a church event. During church services, congregant sing and worship loudly, sit close to each other and often touch surface/fomite which may be contaminated. COVID-19 has proven to be highly contagious and transmitted mainly by droplets or close contact with asymptomatic carriers and infected persons [13]. Hence experts have cautioned that extreme precaution must be taken when dealing with an infected person [14]. In the Free state, three church leaders have since tested positive after leading the church prayer service. Other church leaders and lay preachers who attend the prayer meeting have also tested positive. To date, over 80% of the Free State COVID emanated from this single religious event leading to the infection of over 80 persons and the further tracing of 1600 people who may have been exposed to the virus [15]. Currently, the total number of patients with COVID-19 in the Free State province is 163 (Figure 1), and the infections have dropped marginally.

Traditional male circumcision is cultural maturation rite from boyhood to manhood and widely practised in many South African cultures [16,17]. However, conducting this activity at this time of COVID-19 is a ticking time bomb. In the Eastern Cape Province, circumcision schools are suspended due to COVID-19. However, some illegal schools continue to operate, risking the lives of many young people. The provincial government recently sent teams to districts such as Chris Hani, Komani, OR Tambo, and Port St John's to rescue boys in illegal schools [18]. Although no case of COVID-19 has been reported from the activities of illegal circumcision schools, we must reiterate the danger of the spread of COVID-19 from such activities.

In conclusion, religious and cultural activities of any form must be restricted at this time. The regulation approving 50 persons per burial presents an opportunity for the spread of COVID-19. Hence, only immediate family members should be allowed to bury their loved ones. The government must intensify the enforcement of lockdown measure and promptly identify miscreants with dubious travel permit documents and those who travel with empty caskets to evade police arrest. The Following guidelines by the WHO must further be strictly implemented such as (i) washing your hands frequently and carefully, especially after contact with infected people or their environment; (ii) avoid touching your face including mouth, nose and eyes; (iii) cover your mouth and nose when coughing and sneezing; (iv) take social distancing seriously by keeping a distance of 2 metres from other people; and (v) self-quarantine if sick and wear a mask when you need medical care [3].
